# Fuzzy species among recombinogenic bacteria

**DOI:** 10.1186/1741-7007-3-6

**Published:** 2005-03-07

**Authors:** William P Hanage, Christophe Fraser, Brian G Spratt

**Affiliations:** 1 Department of Infectious Disease Epidemiology, Imperial College London, London, UK

## Abstract

**Background:**

It is a matter of ongoing debate whether a universal species concept is possible for bacteria. Indeed, it is not clear whether closely related isolates of bacteria typically form discrete genotypic clusters that can be assigned as species. The most challenging test of whether species can be clearly delineated is provided by analysis of large populations of closely-related, highly recombinogenic, bacteria that colonise the same body site. We have used concatenated sequences of seven house-keeping loci from 770 strains of 11 named *Neisseria *species, and phylogenetic trees, to investigate whether genotypic clusters can be resolved among these recombinogenic bacteria and, if so, the extent to which they correspond to named species.

**Results:**

Alleles at individual loci were widely distributed among the named species but this distorting effect of recombination was largely buffered by using concatenated sequences, which resolved clusters corresponding to the three species most numerous in the sample, *N. meningitidis*, *N. lactamica *and *N. gonorrhoeae*. A few isolates arose from the branch that separated *N. meningitidis *from *N. lactamica *leading us to describe these species as 'fuzzy'.

**Conclusion:**

A multilocus approach using large samples of closely related isolates delineates species even in the highly recombinogenic human *Neisseria *where individual loci are inadequate for the task. This approach should be applied by taxonomists to large samples of other groups of closely-related bacteria, and especially to those where species delineation has historically been difficult, to determine whether genotypic clusters can be delineated, and to guide the definition of species.

## Background

The definition of bacterial species, and a concept of species applicable to all bacteria, are problems that have long exercised systematists and microbiologists [1-4]. While species names have been assigned to groups of organisms sharing many common phenotypic traits, and a certain minimum level of genomic similarity, attempts to define species using DNA sequences have been relatively unsuccessful. The existence of very different levels of sequence diversity among named species, and the variable extent of gene flow within and between bacterial taxa [5], complicates species concepts and definitions. Indeed, for many, bacterial species are constructs of the human mind, arising from our desire to impose order on the bacterial kingdom [6,7], rather than natural subdivisions imposed by underlying genetic processes, and a central question is not so much how species should best be assigned, but whether such entities exist and can be delineated.

Molecular approaches to assigning bacteria to species began with the introduction of DNA-DNA hybridization, which allowed an objective assessment of the extent of sequence similarity among a set of genomes, and remains the systematicist's gold standard, defining bacterial species as those isolates whose genomes show at least 70% hybridization under standardized conditions [3]. However, few laboratories now use this method and, in practice, novel isolates (and particularly those that presently are unculturable) are usually compared to each other, and to known taxa, by assessing the sequence similarities in their 16S rRNA genes. 16S rRNA sequences are highly conserved and do not provide sufficient resolution to explore the relatedness among closely related bacterial populations and less conserved genes need to be used to delineate similar species.

Individual isolates of a named species differ in gene content [8] and the distribution of these genes is key to understanding the variable properties of isolates of a species, particularly among bacterial pathogens. These auxiliary loci exist alongside the set of genes that are present in all isolates of the named species (the core genome) and which include those that encode enzymes with house-keeping functions [9]. Besides being present in all isolates of a species, the genetic variation in core house-keeping genes is considered to be largely neutral, and thus provides a more reliable indication of genetic relatedness than variation in genes that are subject to strong selection [10]. We would expect any reasonable definition of a species to delineate a cluster of isolates that have very closely-related house-keeping loci that are present in all isolates of a species (as has also been proposed for eukaryotes [11]). However, single house-keeping loci are unlikely to have sufficient variation to allow confident resolution of the different lineages. For recombinogenic bacteria, and arguably all bacteria, multi-locus approaches are required, as these provide increased resolution, and also reduce the impact of 'inter-species' recombination. Thus, a localized interspecies recombinational event at one locus, which distorts the true relatedness between species, is buffered by the more reliable indications of relatedness provided by the other loci. Furthermore, attempts to observe whether or not species exist, and how sharply they can be defined, requires the analysis of large populations of each candidate species and not just one or a few reference isolates.

A multilocus approach has recently been applied to small numbers of isolates of several relatively distantly related named species of enterobacteria [10], and other bacteria [11], and to larger numbers of isolates of related bacteria that are believed to have relatively low rates of recombination [12-14]. However, it is unclear whether species can be resolved using a multilocus approach in the more challenging case of highly recombinogenic bacteria colonising the same body site. Ideally, we would like to know if, in a large collection of such isolates that are believed to include examples of a number of closely related named species, we can resolve well delineated clusters, and the extent to which any clusters relate to the species names assigned by standard microbiological procedures. Can such populations diverge into distinct populations, and stay distinct, in the face of frequent and promiscuous recombination?

In this study we have evaluated the ability of seven individual house-keeping gene sequences, and of the concatenated sequences of these genes, to resolve a large sample of human pathogenic and commensal *Neisseria *into genotypic clusters. We chose this example because *Neisseria *are naturally transformable, are among the most recombinogenic bacteria, and there is good evidence for relatively frequent localised recombination between the named *Neisseria *species [15,16] through transformation. We demonstrate that individual genes are incapable of identifying consistent clusters among the *Neisseria *isolates, but the tree based on the concatenated sequences effectively resolves the three major named species within the sample, although the boundaries are fuzzy due to the presence of a small number of intermediate genotypes.

## Results

**Figure 1:**
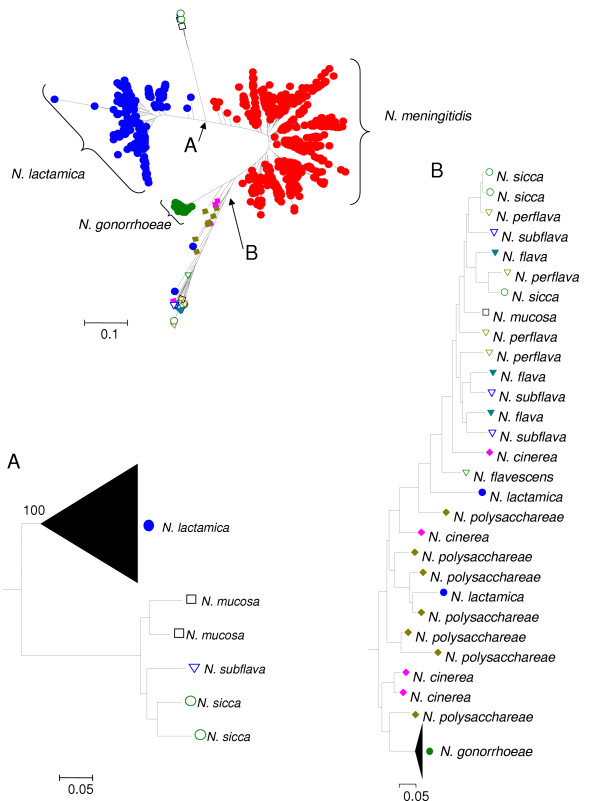
**A tree constructed from concatenated MLST data resolves species clusters in *Neisseria*. **The 50% majority rule consensus tree shown was produced in Mr Bayes using third position sites within the concatenated sequences of *Neisserial *species as described in methods. Assigned species names are indicated by the symbols. Two subtrees, A and B, are shown. These both contain strains identified as *N. mucosa*, *N. sicca *and *N. subflava*, showing that these named species do not form single clusters by this method. In these subtrees, the clades containing exclusively *N. lactamica *or *N. gonorrhoeae *have been collapsed.

The widespread use of multilocus sequence typing (MLST) [17] for epidemiological purposes provides the sequences of seven house-keeping gene fragments from thousands of isolates of several bacterial pathogens. However, few of the available MLST databases include any substantial numbers of isolates of multiple closely related named species. An exception is the public *Neisseria *MLST database, which includes several thousand sequence types (STs) of *N. meningitidis *and smaller numbers assigned to several other named human *Neisseria *species [18] on the basis of standard phenotypic tests. The first 500 STs of *N. meningitidis *were compared with all STs assigned to the other human *Neisseria *species. The sequences of the seven gene fragments were concatenated in-frame and a tree was constructed (using third codon position sites) using Mr Bayes [19]. Figure 1 is the majority rule consensus of 10 000 trees generated from the posterior probability at stationarity. All 67 STs of *N. gonorrhoeae*, and all but two of the 171 STs of *N. lactamica*, descend from single well-supported nodes (the remaining two *N. lactamica *clustered very anomalously and have probably been incorrectly identified). The great majority of *N. meningitidis *also formed a single well-resolved cluster, but a few arise from the branch leading to the *N. lactamica *isolates. Very similar clustering of these three species was observed using other sets of 500 *N. meningitidis *STs from the database, and in a neighbour-joining tree constructed using all STs in the *Neisseria *MLST database (data not shown). The high levels of recombination in the *Neisseria *make the fine structure of the tree meaningless (Figure 1), and here we use the tree-building software first and foremost as a clustering tool.

**Figure 2:**
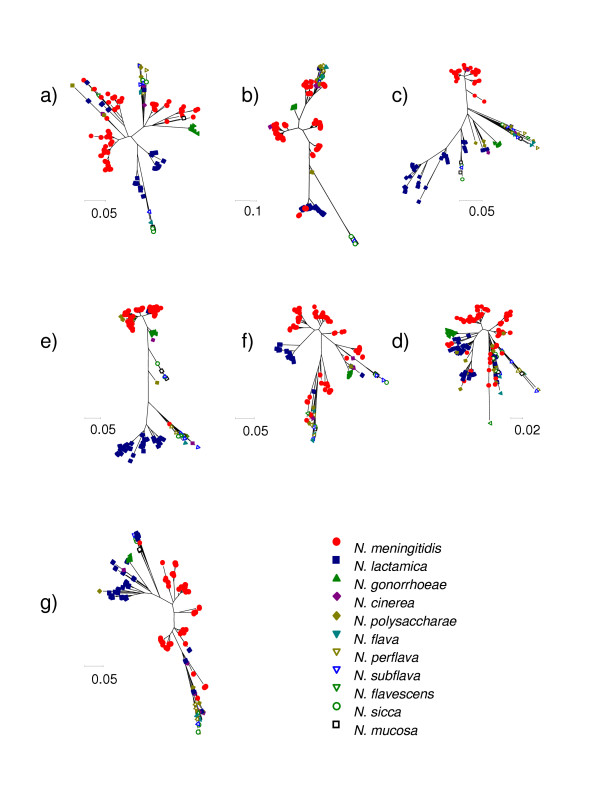
**Single locus trees fail to resolve species clusters. **Individual minimum evolution trees constructed in Mega 2.1 for a) *abcZ*, b) *adk*, c) *aroE*, d) *fumC*, e) *gdh*, f) *pdhC *and g) *pgm*. The assigned species names of the strains in which these alleles occur are shown.

Analysis of the individual gene trees shows that these fail to resolve the named species and highlights many examples where interspecies recombination has resulted in anomalous clustering (Figure 2). The clear inability of single locus trees to resolve the named species, which are well resolved using the concatenated sequences, establishes that multiple loci are required to buffer against the distorting effect of inter-species recombination at the individual loci. Although the concatenated sequences resolve three named species, *N. gonorrhoeae*, *N. meningitidis *and *N. lactamica*, their boundaries are not perfectly defined and a number of isolates are placed on the branch between *N. lactamica *and *N. meningitidis*, representing intermediate genotypes.

The small numbers of STs assigned to other human *Neisseria *species do not cluster clearly. A significant separation is observed between two subtrees (A and B in Figure 1), although these both contain isolates assigned as *N. sicca*, *N. mucosa *and *N. subflava*. Multiple minimum-evolution trees constructed using all STs of these other *Neisseria *species and randomly selected samples of ten STs from each of *N. meningitidis*, *N. lactamica *and *N. gonorrhoeae*, showed the same deep split between these subtrees, which was also observed in trees constructed from all *Neisseria *STs (all species) in the MLST database, using Neighbour-Joining, minimum evolution and UPGMA tree-building approaches (data not shown).

## Discussion

Current molecular definitions of species use rules or cut-off values (e.g. ≥ 70% DNA-DNA hybridization) and rarely take account of the genotypic diversity within and between populations [3]. A more natural and pragmatic approach is to analyse large populations of related isolates, that are believed to cover multiple species, and to observe whether suitable molecular methods can resolve distinct clusters in sequence space that can be given appropriate names [11]. This approach has not yet been rigorously applied to bacteria. Consequently we have no idea whether large populations of related bacteria can invariably be divided into discrete clusters using suitable molecular methods or, alternatively, whether many groups of related bacteria fall into a genetic continuum where clear divisions do not exist.

Sequence-based approaches should help us answer this question. However, most studies have focused on single loci and small numbers of isolates, whereas multilocus approaches with large populations are essential as the history of individual genes (including rRNA operons [20]) may be obscured by interspecies recombination, and clusters observed using a small number of isolates may merge when larger numbers of isolates are considered. Comparison of the tree based on the concatenated sequences with the individual gene trees clearly illustrates the inadequacy of single loci for resolving *N. meningitidis *and *N. lactamica *(Figure 2). The concatenation of the seven housekeeping loci shows that multiple loci can buffer against the distorting effects of inter-species recombination and that the boundaries between the three dominant species in the *Neisseria *MLST database can be resolved.

Network based methods (e.g. Neighbor-Net [21], Splitstree [22]) applied to both the concatenates and individual loci produce output with numerous reticulations, indicating the conflicting signals in the data, such that the implied relationships between STs within clusters have no phylogenetic meaning. Nevertheless, the use of multiple loci enables us to observe the species clusters even in the presence of conflicting signals. The three main clusters coincide well with the species names derived by standard microbiological procedures and the present definitions of *N. meningitidis*, *N. lactamica *and *N. gonorrhoeae *are reasonably secure; the two *N. lactamica *that clustered highly anomalously probably represent species mis-identification. The most critical test of the multilocus approach is the ability to resolve *N. lactamica *from *N. meningitidis *since these colonise the same body site, the nasopharynx. Resolution of these named species was remarkably good, although the boundaries between *N. lactamica *and *N. meningitidis *are somewhat fuzzy, due to the existence of intermediate forms. This is to be expected as recombinogenic bacteria have mosaic genomes, resulting from the occasional replacement of chromosomal segments with those from related populations. Thus, in any large dataset, there may be isolates in which one or more of the loci used in a multilocus approach to species definition will have been recently introduced from a related population. Single unusually divergent replacements, or replacements at more than one of the multiple loci, may place isolates away from the majority of isolates of the species. However, only seven STs in Figure 1 fell into this category (of 667 STs from isolates identified as either *N. meningitidis *or *N. lactamica*), and there was no overlap between these two named species (i.e. a region containing isolates identified as both species interspersed with one another).

Sorting the human commensal *Neisseria *into species has been difficult, with frequent revisions of species names [23]. We gain some insight into the extent and source of this difficulty in Figure 1, where isolates assigned as *N. mucosa*, *N. sicca *and *N. subflava *each fall in very different parts of the tree, and the subtree shown in Figure 1A contains several closely related isolates that have been assigned to these three different named species. Additional studies of the human commensal, *Neisseria *(and of other groups plagued with similar problems, such as viridans streptococci) using the multilocus approach with large datasets, should clarify whether they fall into distinct clusters, or whether the difficulties in defining species by phenotypic methods reflect an underlying genetic reality in which resolved clusters are not evident.

If necessary, further resolution between apparent clusters may be attempted by increasing the numbers of loci sequenced. Provided that the alleles at these loci show a degree of specificity to a given species cluster, then the resolution of that cluster will be enhanced. If this cannot be demonstrated, then it is likely that the isolates under test do not genuinely form separate populations, and should not be considered to be distinct species. This approach lends itself to "electronic taxonomy", in which systematic classification may be evermore finely elucidated through the accumulation of online sequence databases.

The work described here obviously begs the question of what forces or mechanisms could generate such separation among recombining bacteria. We offer a simple model for recombining organisms as follows: consider two populations freely recombining within themselves and with each other. New mutations arising in one population will readily spread to the other, and to an observer they appear to form one cluster of related strains. If a barrier to recombination should be erected between them, such that isolates are much more likely to undergo recombination with their own population, then the rate of generation of new genotypes within each population may increase beyond the rate at which such genetic innovation is shared and the two populations begin to diverge. As the populations diverge, decreasing sequence identity will further impede recombination, thus reinforcing the effect of the original genetic barrier and creating a permanent separation [24,25].

It is not difficult to suggest candidate mechanisms. Niche separation is one example, and almost certainly underlies the tight well-defined cluster of *N. gonorrhoeae*. Unlike the other human *Neisseria*, which colonise the nasopharynx, the primary niche of the gonococcus is the genital tract, and it has been proposed that gonococci arose relatively recently due to the successful invasion of the genital tract by a nasopharyngeal *Neisseria *lineage [26]. Similarly, what appears to be single body site (e.g. the human nasopharynx) may contain multiple niches that can be exploited, leading to opportunities for speciation. Restriction-modification systems [27], limitation of transformability by differences in pheromone-type [28] and similar processes are feasible alternatives.

The point at which such a group is described as a species is a matter more of human interest and attention than any intrinsic evolutionary process. The properties of the species clusters we observe will be determined by the diversification of those strains sharing the speciation loci (i.e those that determine gene flow). Because speciation is gradual, we should be able using estimates of recombination within and between groups derived from multilocus data, to define nascent species which if they continue to diversify in isolation, are expected to form distinct sequence clusters, ie species, in the future.

## Conclusion

The bacterial domain of life is not uniform. Instead we see clumps of similar strains that share many characteristics, and with an innate human urge to classify, we have defined these as species. This work shows that by applying a simple approach using sequence data from multiple core housekeeping loci, we can resolve those clusters, provided such clusters exist. However, these species clusters are not ideal entities with sharp and unambiguous boundaries; instead they come in multiple forms and their fringes, especially in recombinogenic bacteria, may be fuzzy and indistinct. A multilocus approach using large numbers of isolates will provide data that help us to develop theoretical models of how species emerge, and relate these to the observed population genetic structure of bacteria. This should be enormously helpful to taxonomists, whose foremost duty will remain to provide us with pragmatic species designations which attempt to reflect the underlying genetic reality.

## Methods

### Strains

The contents of the publicly accessible *Neisseria *MLST database [17,18] were used to explore the validity of the approach described here for other species. Alleles at the seven MLST loci of all isolates defined as Neisserial species other than *N. meningitidis *(67 isolates of *N. gonorrhoeae*, 171 of *N. lactamica*, 5 of *N. sicca*, 3 of *N. mucosa*, 5 of *N. cinerea*, 7 of *N. polysacchareae*, 3 of *N. flava*, 4 of *N. perflava*, 4 of *N. subflava *and 1 isolate of *N. flavescens*) were concatenated as described below, and analysed together with the concatenated sequences of *N. meningitidis *strains with ST numbers from 1 to 500. Species definitions were as recorded at [17,18], and were according to standard clinical microbiological schema. The sequences of the individual alleles at the seven loci in the above *Neisseria *were also used to construct individual gene trees.

### Phylogenetics and population genetics

MLST loci were concatenated in-frame to form a 3267 bp sequence, of which only third position sites were used in subsequent analyses. To illustrate clustering in this dataset, a tree was constructed using Mr Bayes 3.0b4 [19]. A starting tree was determined in PAUP (version 4 beta 10) [29] using the Neighbour-Joining method with distances corrected using the HKY85 model. The starting tree was input into Mr Bayes, and four Markov Chain Monte Carlo chains were run with default heating parameters until convergence and 10 000 trees were sampled from the posterior probability distribution. These were then used to produce a 50% majority rule consensus tree. Minimum evolution trees for individual loci were constructed in MEGA 2.1 [30]. Third position sites were used with the Kimura 2-parameter distance correction.

## List of abbreviations

rRNA ribosomal RNA

MLST Multi Locus Sequence Typing

ST Sequence Type

UPGMA Unweighted Pair Group Method with Arithmetic Mean

## Authors' contributions

BGS conceived of the study and drafted the manuscript, CF participated in study design and analysis of results, WPH designed the study, carried out the analyses and interpreted the results, and drafted the manuscript.
